# Is TikTok increasing the number of self-diagnoses of ADHD in young people?

**DOI:** 10.1192/j.eurpsy.2022.1463

**Published:** 2022-09-01

**Authors:** R. Gilmore, J. Beezhold, V. Selwyn, R. Howard, I. Bartolome, N. Henderson

**Affiliations:** University of East Anglia, Norwich Medical School, Norwich, United Kingdom

**Keywords:** TikTok, e-mental health, adhd, Child and adolescent psychiatry

## Abstract

**Introduction:**

TikTok is a free mobile application, that enables users to create short videos. TikTok has an estimated one billion monthly active users, comprised of a mostly younger audience. There has been a noticed rise in content discussing ADHD – hashtag ADHD on TikTok has 6.3 billion views. The discussions continue on Twitter, where users are reporting watching TikTok content explaining ADHD symptomatology, subsequently relating to the condition and requesting referrals to specialist psychiatry services. This study aims to identify key themes in discussions around TikTok and ADHD, and its ramifications.

**Objectives:**

This study’s objective is to discuss the relationship between viewing ADHD content of TikTok and self-diagnoses of ADHD in young people.

**Methods:**

In our study, Twitter posts were identified with the words ‘ADHD’ and ‘TikTok’ and established key themes relating to self-diagnosis of ADHD.

**Results:**

Numerous tweets were found discussing individual’s experiences of self-diagnosis of ADHD after watching TikTok videos and relating with the symptomology. Furthermore, many users discussed their efforts to seek diagnosis from psychiatrists. These posts highlighted positive discussion of mental health, and the improvement in quality of life since diagnosis.

**Conclusions:**

Many young people are self-diagnosing ADHD after viewing TikTok videos. This may improve mental health stigma, however the expertise of the video creators should be scrutinised. Furthermore, the impact on already stretched waiting lists should be considered, with individuals who’s perceived ADHD traits are not impacting on their quality of life.

**Disclosure:**

No significant relationships.

